# Benefits of Rhythmic Auditory Stimulation and Lee Silverman Voice Treatment in a Parkinson’s Disease Patient With Meningoencephalitis: A Case Report

**DOI:** 10.7759/cureus.72555

**Published:** 2024-10-28

**Authors:** Aditi Dandekar, Pallavi Harjpal, Disha S Songire

**Affiliations:** 1 Department of Neurophysiotherapy, Ravi Nair Physiotherapy College, Datta Meghe Institute of Higher Education & Research, Wardha, IND

**Keywords:** lsvt, parkinson’s disease, ras, rehabilitation, viral meningoencephalitis

## Abstract

Parkinson’s disease (PD) is a neurological disorder characterized by motor and non-motor symptoms that significantly impact patients’ quality of life. Meningoencephalitis, an inflammation of the brain and its surrounding membranes, exacerbates the neurological features of PD, leading to considerable disability. This case report describes a 73-year-old female with a five-year history of PD who presented with meningoencephalitis. She exhibited pyrexia, dyspnea, and neurological symptoms consistent with meningeal irritation. Diagnostic investigations indicated viral meningoencephalitis. Following medical management, the patient completed a four-week physical therapy program, including Lee Silverman voice treatment, rhythmic auditory stimulation, and proprioceptive neuromuscular facilitation exercises. These physiotherapy interventions focused on mobilization to improve range of motion, muscle strength, neuromuscular coordination, and functional ability. Quantitative outcome measures - such as the Unified Parkinson’s Disease Rating Scale, Modified Hoehn and Yahr stage, and Parkinson’s Disease Questionnaire-39 - showed positive changes following the intervention. The outcomes highlight the benefits of a multifaceted rehabilitation approach for patients with PD and concurrent meningoencephalitis. This case underscores the importance of initiating physiotherapy early to enhance motor outcomes and quality of life in patients with complex neurological conditions.

## Introduction

Parkinson’s disease (PD) is a prevalent, progressive neurological condition that causes significant motor impairment, leading to daily life challenges and decreased quality of life [1]. It is one of the most common neurodegenerative disorders, affecting around 0.5-1% of adults over 65 and rising to 1-3% in those older [2]. Due to an aging global population, the PD burden is projected to increase by over 30% by 2030, placing a substantial economic strain on societies [3]. Epidemiological data also indicate that men are twice as likely as women to develop PD [4]. Pathologically, PD is marked by the loss of dopamine-producing neurons in specific brain areas, although other cells in different regions are also affected. While the etiology of PD remains idiopathic [5], rare familial cases have been linked to genetic mutations, and environmental factors - such as smoking, caffeine intake, and pesticide exposure - may play a role, although their precise contributions are still under investigation [5].

Clinically, PD manifests through motor symptoms like tremors, bradykinesia, rigidity, and postural instability [2]. Other related motor symptoms may include reduced facial expression (hypomimia), speech difficulties (dysarthria), swallowing challenges (dysphagia), excessive saliva (sialorrhea), small handwriting (micrographia), shuffling gait, short rapid steps (festination), freezing of gait episodes, muscle cramps (dystonia), and an exaggerated blinking reflex (glabellar reflexes). PD is also associated with non-motor symptoms, including autonomic dysfunction, cognitive/neurobehavioral issues, sleep disturbances, tingling sensations (paresthesia), and anosmia [6]. Since PD is often diagnosed in later stages when significant neuronal damage has already occurred, early detection is crucial. Biomarkers derived from imaging, CSF, and blood metabolomic profiles could offer early diagnostic potential, targeting factors such as oxidative stress, α-synuclein clearance, neuroprotection (e.g., nerve growth factor), and inflammatory markers like NFκβ and TNF [7].

Meningoencephalitis, an inflammatory condition affecting the meninges and brain, can be caused by various infectious agents, including viral pathogens like Herpes simplex virus, fungi such as *Cryptococcus neoformans*, parasites, and bacteria like *Streptococcus pneumoniae*, *Haemophilus influenzae*, and *Mycobacterium tuberculosis *[8]. Meningoencephalitis has high morbidity and mortality rates [9], and timely diagnosis and targeted treatment are essential to improve survival and reduce neurological complications [10,11].

Physical therapy plays a crucial role in PD management [12]. It focuses on improving posture, balance, gait, and physical activity through cueing techniques (visual, auditory, and tactile) and cognitive-motor schema strategies. Interventions include personalized exercises to enhance independence, safety, and quality of life [13]. The body of evidence supporting rehabilitation in PD has grown significantly in recent years [14]. For meningoencephalitis, physical therapy aims to restore mobility, increase functional independence, alleviate symptoms, and enhance quality of life. In summary, incorporating physiotherapy alongside medical treatment in meningoencephalitis management is beneficial, facilitating improved function and more favorable rehabilitation outcomes.

## Case presentation

The patient was a 73-year-old female with a diagnosis of PD for the past five years and a history of hypertension over the same duration. Upon hospital admission, she presented with several significant complaints, including fever persisting for five consecutive days, respiratory difficulties for two days, a cough producing yellowish sputum, nausea, and lethargy. Her vital signs at the time of admission indicated fever, hypertension, tachypnea, and probable hypoxemia.

A neurological assessment revealed behavioral changes and symptoms indicative of meningeal irritation. To investigate these complaints, a lumbar puncture and complete blood count (CBC) were performed. The lumbar puncture results demonstrated predominant mononuclear cell infiltration, leading to a diagnosis of meningoencephalitis. The serological tests, particularly the CBC, indicated a mild elevation in WBC count at 10,800 cells/microliter, suggestive of a viral etiology for meningoencephalitis.

Further investigations included antibiotic sensitivity tests, blood cultures, and sputum cultures to isolate the various potential causative organisms. The patient was initiated on antibiotics effective against meningoencephalitis and received antipyretics to manage her fever. Additionally, symptomatic medications were prescribed in light of her altered sensorium and swallowing difficulties.

In regard to her Parkinson’s management, her PD medications were adjusted as necessary. This included administering Pacitane 2 mg three times a day and Syndopa 110 mg twice daily. Her hypertensive condition was managed with amlodipine 5 mg and prazosin 1 mg, both given once daily.

Clinical findings

Consent was obtained from the patient prior to commencing the assessment. During the general evaluation, the patient was fully alert, cooperative, and oriented to person, place, and time. She was hemodynamically stable. The neurological examination revealed cogwheel rigidity (Grade 3 - marked, but full range of motion was achieved easily) in both upper and lower extremities, along with prominent resting tremors in both upper limbs. Bilateral reflexes were exaggerated, and the plantar response was withdrawn. Sensations were intact on both sides.

Timeline

The patient was admitted to the neurology ward in July 2024. Her hospitalization details are documented in Table 1.

**Table 1:** Timeline of patient’s hospital stay

Timeline	Date
Date of admission	July 28, 2024
Date of diagnostic investigations	July 29, 2024
Date of physiotherapy rehabilitation session	August 1, 2024
Date of discharge	August 27, 2024

Physiotherapy interventions

The physiotherapy regimen provided to the patient for four weeks in the inpatient department is detailed in Table 2 [8,12,14].

**Table 2:** Physiotherapy interventions provided to the patient LSVT: Lee Silverman voice treatment, PNF: proprioceptive neuromuscular facilitation

Physiotherapy goals	Physiotherapy interventions	Dosage regimens
To normalize the muscle tone	Roods inhibitory approach including icing and sustained stretched	7-10 minutes, twice per day
To improve relaxation	Rocking techniques	15-30 repetitions
To improve the psychological domain	Meditation	15 minutes, twice per day
To prevent muscle stiffness and soreness	Stretching exercises	Two to three times a day, up to 15 minutes each session
To improve joint flexibility	Passive range of motion exercises	2-3 sets of 10-12 repetitions with 5- to 10-second hold
For enhanced neuromuscular coordination	PNF exercises (D1-D2 flexion-extension pattern); Techniques used: rhythmic initiation and rhythmic stabilization	10-12 repetitions, 2-3 sets per session, 2-3 times weekly
For the improvement of muscle strength and functional outcomes in the extremities and trunk	Strength training using Delorme’s protocol [12]	2-4 sets, 8-10 repetitions for a week
To enhance the contraction of the facial muscles	Facial PNF exercises, facial massage	20 repetitions, 2 sets
To enhance the general amplitude of movements and basic motor coordination	LSVT BIG approach [15]	16 sessions, 4 days a week
For enhancement of vocal loudness and clarity	LSVT LOUD approach [16]	16 sessions, 4 days a week
For improving gait speed and synchronizing it with the rhythm and for better motor skills, initiation, and execution of movements	Rhythmic auditory stimulation, including synchronization exercises with rhythmic cues and gait training with rhythmic auditory cues [17]	Three to four sessions per week each, each session being 30 minutes

**Figure 1:**
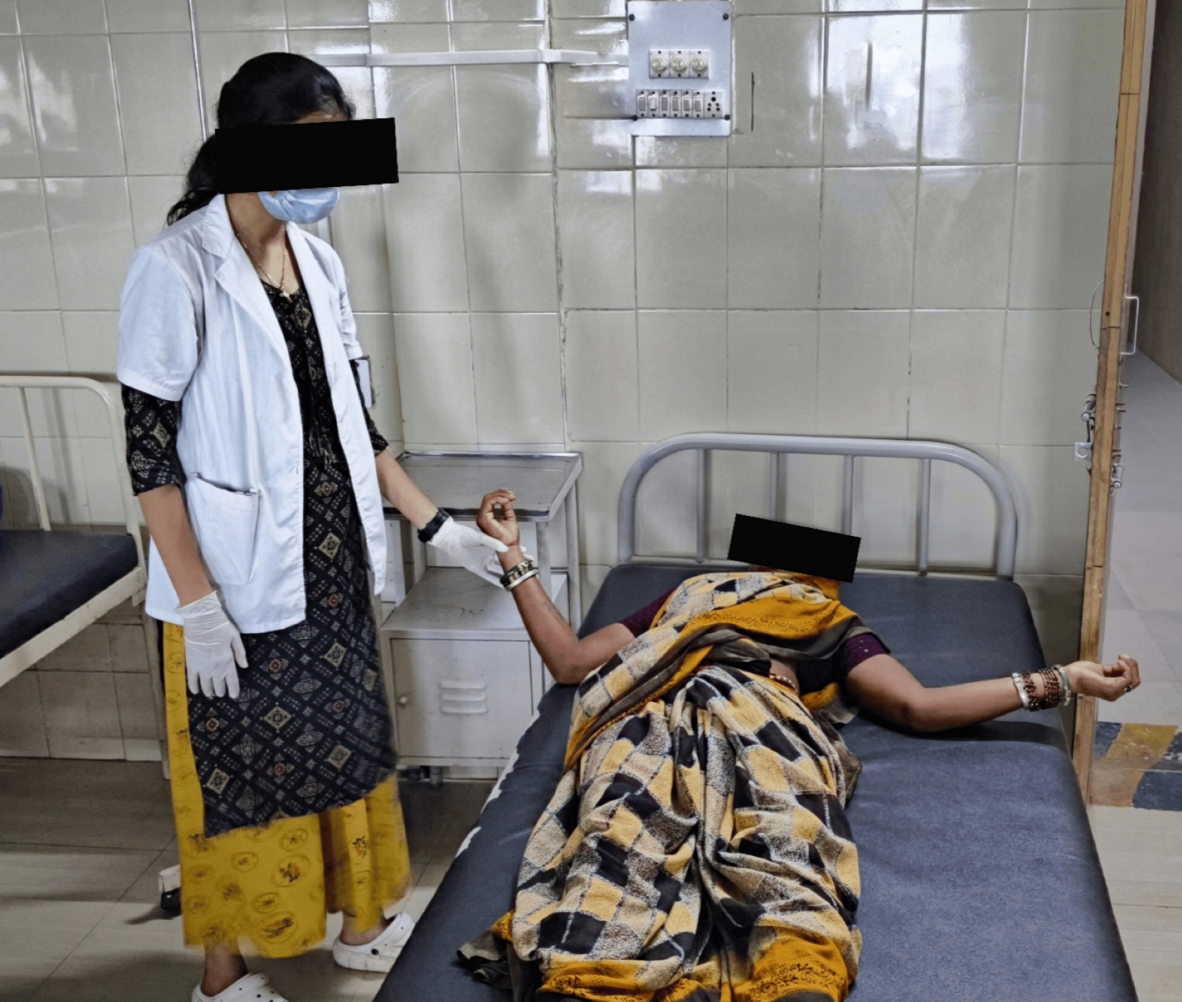
Patient performing LSVT exercise in the supine position LSVT: Lee Silverman voice treatment

Figure 1 illustrates the patient performing LSVT BIG in a supine position, which emphasizes improving movement amplitude. This approach assists the patient in moving more freely and enhances the ability to perform daily tasks with greater ease.

Outcome measures

Table 3 summarizes the reflex examination results, highlighting significant improvements from pre- to post-intervention over four weeks. Prior to the intervention, reflexes were noted to be exaggerated (+++), while post-intervention assessments revealed responses within the normal range (++), indicating that all reflexes returned to normal function.

**Table 3:** Pre- and post-intervention reflex examination + = Diminished reflex, + + = Normal reflex, + + + = Exaggerated reflex, ++++ = Hyperactive with clonus reflex

Reflexes	Pre-intervention		Post-intervention	
	Right	Left	Right	Left
Biceps reflex	_+ + +_	_+ + +_	_+ +_	_+ +_
Triceps reflex	_+ + +_	_+ + +_	_+ +_	_+ +_
Supinator reflex	_+ + +_	_+ + +_	_+ +_	_+ +_
Knee reflex	_+ + +_	_+ + +_	_+ +_	_+ +_
Ankle reflex	_+ + +_	_+ + +_	_+ +_	_+ +_
Plantar response	Withdrawal	Withdrawal	Flexor	Flexor

Table 4 displays the results of the tone examination conducted pre- and post-intervention using the Tone Grading Scale [18]. Prior to the intervention, muscle tone on both sides was assessed at “3+,” indicating increased tone. However, following the four-week intervention, a notable improvement was observed, with muscle tone decreasing to a normal “2+” on each side of the body, indicating relaxation.

**Table 4:** Pre- and post-intervention tone examination 0: No tone, 1+: Hypotonia, 2+: Normal tone, 3+: Mild to moderate hypertonia, 4+: Severe hypertonia

Muscles	Pre-intervention		Post-intervention	
	Right	Left	Right	Left
Shoulder	3+	3+	2+	2+
Elbow	3+	3+	2+	2+
Wrist	3+	3+	2+	2+
Hip	3+	3+	2+	2+
Knee	3+	3+	2+	2+
Ankle	3+	3+	2+	2+

The outcome measures demonstrated significant improvement following the intervention, as detailed in Table 5. Prior to the intervention, the patient had a Unified Parkinson’s Disease Rating Scale score of 111 out of a possible 260, indicating a considerable level of disability (validity: 0.81, reliability: 0.85, minimal clinically important difference (MCID): 4-8). After the intervention, the score improved to 190 out of 260, reflecting enhanced overall function. Additionally, the patient’s progression in PD was assessed using the Modified Hoehn and Yahr Staging Scale, which indicated a change from Stage 5, representing severe disability, to Stage 2.5, indicating moderate disability (validity: 0.92, reliability: 0.85, MCID: -1 to +1). Furthermore, the patient’s quality of life showed marked improvement, as evidenced by a reduction in the Parkinson’s Disease Questionnaire-39 score from 69.87% at baseline to 37.18% post-intervention (validity: 0.94, reliability: 0.87, MCID: -4.72 to +4.22).

**Table 5:** Outcome measures

Outcome measures	Pre-intervention	Post-intervention
Unified Parkinson’s Disease Rating Scale	111/260	190/260
Modified Hoehn and Yahr Staging Scale	Stage 5	Stage 2.5
Parkinson’s Disease Questionnaire-39	69.87%	37.18%

## Discussion

PD is a major degenerative neurological disorder where motor functions deteriorate progressively, making even basic movements extremely challenging [6]. Meningoencephalitis, a severe neurological condition involving inflammation of the brain and surrounding tissues, typically arises from viral, bacterial, or fungal infections [8]. This case report highlights the need for a multimodal rehabilitation approach to manage PD patients with concurrent meningoencephalitis, as the combination of these conditions exacerbates disability and demands individualized rehabilitation strategies. Physical therapy is essential for both conditions, as it promotes mobility, balance, and functional abilities. However, when meningoencephalitis co-occurs with PD, rehabilitation goals must be adapted to address the neurological complications introduced by brain inflammation.

Lee et al. emphasize that managing parkinsonism requires a comprehensive strategy involving medical, surgical, and rehabilitative treatments. While no definitive cure exists, the aim is to enhance patient quality of life and functional abilities through well-defined treatment plans [19]. Combining a multifaceted treatment strategy with ongoing research into new therapies could offer more effective management and improve the quality of life of those affected by PD.

For PD, rehabilitation should be “goal-based,” focusing on mastering core skills tailored to patient needs. The program must also consider practice variables such as intensity, specificity, and complexity, as highlighted by Abbruzzese et al. [20]. A 2020 meta-analysis by Radder et al. provides a comprehensive overview of the evidence supporting various physiotherapy interventions for PD, helping individuals and clinicians select effective treatments [12]. Pélissier and Pérennou suggest that outpatient units incorporating medical and rehabilitation services are particularly suitable for assisting patients with PD in managing functional limitations and promoting self-care [21].

Bleton’s research explores cognitive strategies for rehabilitation, such as task rehearsals, to alleviate akinesia, a hallmark of parkinsonism [22]. Peterka et al. found that LSVT-BIG training positively influences proprioceptive processing in PD patients [15]. Thaut et al. demonstrated that patients training with rhythmic auditory stimulation (RAS) significantly improved gait velocity, stride length, and cadence [17]. According to McDonnell et al., LSVT-BIG improves motor function more effectively than general exercises, providing moderate-quality evidence supporting amplitude-oriented training to reduce motor disabilities in PD patients with mild disease severity [23].

Research by Ye et al. shows that RAS enhances motor aspects like gait, mobility, and quality of life for PD patients [24]. Forte et al. observed positive effects on gait and mobility through rhythmic auditory cueing with exercise, recommending it as part of routine rehabilitation for PD patients [25]. Eldemir et al. reported that a modified LSVT-BIG protocol showed promising results in balance and gait, offering a flexible treatment option for patients who struggle with the frequency requirements of the standard protocol [26].

## Conclusions

This case illustrates the benefits of a comprehensive rehabilitation intervention employing diverse approaches to treat a patient with PD complicated by meningoencephalitis. Through individualized and multimodal physiotherapy interventions, the patient’s motor function and overall quality of life improved significantly. The physiotherapy regimen played a crucial role in achieving these outcomes, addressing the multiple motor manifestations associated with both PD and meningoencephalitis.

The successful integration of intervention strategies, such as LSVT BIG, proprioceptive neuromuscular facilitation, strength training, and RAS, enhanced mobility, neuromuscular coordination, muscle tone, and balance. Physical therapy not only facilitated the recovery of the patient’s functional mobility but also alleviated symptoms and increased functional capacity. These findings emphasize the importance of timely and continuous physiotherapy for managing complex neurological conditions, ultimately enhancing the patient's quality of life.

## Appendices

PDQ-39 QUESTIONNAIRE

Due to having Parkinson’s disease, how often during the last month

Please tick one box for each question

have you....                                          Never      Occasionally      Sometimes   Often    Always or cannot do at all

1              Had difficulty doing the leisure activities which you would like to do?

2              Had difficulty looking after your home, e.g. DIY, housework, cooking?

3              Had difficulty carrying bags of shopping?

4              Had problems walking half a mile?

5              Had problems walking 100 yards?

6              Had problems getting around the house as easily as you would like?

7              Had difficulty getting around in public?

8              Needed someone else to accompany you when you went out?

9              Felt frightened or worried about falling over in public?

10            Been confined to the house more than you would like?

11            Had difficulty washing yourself?

12            Had difficulty dressing yourself?

13            Had problems doing up your shoe laces?

Due to having Parkinson’s disease, how often during the last month

Please tick one box for each question

have you....                                          Never     Occasionally     Sometimes     Often      Always or cannot do at all

14            Had problems writing clearly?

15            Had difficulty cutting up your food?

16            Had difficulty holding a drink without spilling it?

17           Felt depressed?

18           Felt isolated and lonely?

19           Felt weepy or tearful?

20           Felt angry or bitter?

21           Felt anxious?

22            Felt worried about your future?

23            Felt you had to conceal your Parkinson's from people?

24            Avoided situations which involve eating or drinking in public?

25            Felt embarrassed in public due to having Parkinson's disease?

26            Felt worried by other people's reaction to you?

27            Had problems with your close personal relationships?

28            Lacked support in the ways you need from your spouse or partner?

If you do not have a spouse or partner tick here

29            Lacked support in the ways you need from your family or close friends?

Due to having Parkinson’s disease, how often during the last month

Please tick one box for each question

have you....                                          Never         Occasionally  Sometimes        Often           Always

30            Unexpectedly fallen asleep during the day?

31            Had problems with your concentration, e.g. when reading or watching TV?             

32            Felt your memory was bad?

33            Had distressing dreams or hallucinations?

34            Had difficulty with your speech?

35            Felt unable to communicate with people properly?

36            Felt ignored by people?

37            Had painful muscle cramps or spasms?

38            Had aches and pains in your joints or body?

39            Felt unpleasantly hot or cold?
